# Unravelling the *Neospora caninum* secretome through the secreted fraction (ESA) and quantification of the discharged tachyzoite using high-resolution mass spectrometry-based proteomics

**DOI:** 10.1186/1756-3305-6-335

**Published:** 2013-11-23

**Authors:** Letícia Pollo-Oliveira, Harm Post, Marcio Luis Acencio, Ney Lemke, Henk van den Toorn, Vinicius Tragante, Albert JR Heck, AF Maarten Altelaar, Ana Patrícia Yatsuda

**Affiliations:** 1 Faculdade de Ciências Farmacêuticas de Ribeirão Preto e Núcleo de Apoio à Pesquisa em Produtos Naturais e Sintéticos (NPPNS), Universidade de São Paulo, Av do Café , s/n, Ribeirão Preto, SP 14040-903, Brazil; 2 Biomolecular Mass Spectrometry and Proteomics, Utrecht Institute for Pharmaceutical Sciences and Bijvoet Centre for Biomolecular Research, Utrecht University, Padualaan 8, Utrecht 3884 CH, The Netherlands; 3 Netherlands Proteomics Centre, Padualaan 8, Utrecht 3884 CH, The Netherlands; 4 Botucatu Institute of Biosciences, UNESP - Univ Estadual Paulista, Distrito de Rubião Jr, s/n, Botucatu, São Paulo 18918-970, Brazil; 5 Division of Heart and Lungs, Department of Cardiology, University Medical Center Utrecht, Utrecht, The Netherlands; 6 Division of Biomedical Genetics, Department of Medical Genetics, University Medical Center Utrecht, Utrecht, The Netherlands

**Keywords:** Mass spectrometry, *Neospora caninum*, Secretome, Shotgun, Relative quantification

## Abstract

**Background:**

The apicomplexan parasite *Neospora caninum* causes neosporosis, a disease that leads to abortion or stillbirth in cattle, generating an economic impact on the dairy and beef cattle trade. As an obligatory intracellular parasite, *N. caninum* needs to invade the host cell in an active manner to survive. The increase in parasite cytosolic Ca^2+^ upon contact with the host cell mediates critical events, including the exocytosis of phylum-specific secretory organelles and the activation of the parasite invasion motor. Because invasion is considered a requirement for pathogen survival and replication within the host, the identification of secreted proteins (secretome) involved in invasion may be useful to reveal interesting targets for therapeutic intervention.

**Methods:**

To chart the currently missing *N. caninum* secretome, we employed mass spectrometry-based proteomics to identify proteins present in the *N. caninum* tachyzoite using two different approaches. The first approach was identifying the proteins present in the tachyzoite-secreted fraction (ESA). The second approach was determining the relative quantification through peptide stable isotope labelling of the tachyzoites submitted to an ethanol secretion stimulus (*discharged* tachyzoite), expecting to identify the secreted proteins among the down-regulated group.

**Results:**

As a result, 615 proteins were identified at ESA and 2,011 proteins quantified at the *discharged* tachyzoite. We have analysed the connection between the secreted and the down-regulated proteins and searched for putative regulators of the secretion process among the up-regulated proteins. An interaction network was built by computational prediction involving the up- and down-regulated proteins. The mass spectrometry proteomics data have been deposited to the ProteomeXchange with identifier PXD000424.

**Conclusions:**

The comparison between the protein abundances in ESA and their measure in the *discharged* tachyzoite allowed for a more precise identification of the most likely secreted proteins. Information from the network interaction and up-regulated proteins was important to recognise key proteins potentially involved in the metabolic regulation of secretion. Our results may be helpful to guide the selection of targets to be investigated against *Neospora caninum* and other Apicomplexan organisms.

## Background

The Apicomplexa phylum includes many parasites that are relevant to human (such as *Plasmodium* and *Toxoplasma*) and veterinary (such as *Babesia*, *Eimeria*, and *Neospora*) health. *Neospora caninum* is the causative agent of neosporosis, a disease that leads to abortion or stillbirth in cattle. Consequently, the worldwide economic losses in the dairy and beef cattle trade vindicate the development of an effective therapeutic strategy for neosporosis control [1-3].

As with all apicomplexan species, *N. caninum* is an obligate intracellular parasite that invades the host cell in a conserved active manner, which involves the release of proteins from phylum-specific secretory organelles and the activation of the parasite invasion motor. These organelles, known as micronemes, rhoptries and dense granules, secrete proteins crucial for apical attachment, moving junction formation, gliding motility, and parasitophorous vacuole formation and establishment [4-6]. Upon contact with the host cell, there is an increase in parasite cytosolic Ca^2+^ which mediates critical events, such as secretion of adhesins, gliding motility, cell invasion, and egress [7-9]. *In vitro*, ethanol is a known trigger of apical organelle secretion by means of Ca^2+^ mobilisation [10].

Because invasion is considered a requirement for pathogen survival and replication within the host, the identification of secreted proteins (secretome) involved in invasion may be useful to reveal interesting targets for therapeutic intervention [8,11].

Previous proteomic studies in *N. caninum* have focused on the identification of proteins in the total extract using two-dimensional (2D) gels with or without immunoblotting [12-17]. Other studies have used 2D-DIGE followed by mass spectrometry (MS) analyses to compare tachyzoite *versus* bradyzoite profiles [18] or wild-type *versus* attenuated isolates [19]. LC-MS/MS, i.e., MS-based proteomics [20], has been employed in three reports: the identification of antigens from an organelle fraction after the generation of monoclonal antibodies [21], the identification of antigens that stimulated bovine CD4 + ve T cells [22] and the identification of proteins from a rhoptry-enriched fraction [23].

In our study, the *N. caninum* secretome was investigated using the following two different approaches: the identification of proteins present in the tachyzoite secreted fraction (also known as ESA) and the relative quantification of the tachyzoite proteome before and after submission to ethanol stimulated secretion (here called *discharged* tachyzoite). For both approaches, we performed state-of-the-art nanoLC-MS/MS based proteomics, employing a decision tree guided MS strategy, which determines the best combination of fragmentation technique and mass analyser based on the physicochemical properties of the precursor peptide [24].

As a result, 615 proteins were identified in the ESA fraction and 2,011 tachyzoite proteins were quantified before and after discharge. We have analysed the overlap between the secreted proteins observed in ESA and the down-regulated proteins from the discharged tachyzoite. Additionally, we searched for putative regulators of the secretion process among the up-regulated proteins and performed a protein interaction prediction analysis.

## Methods

**Figure 1:**
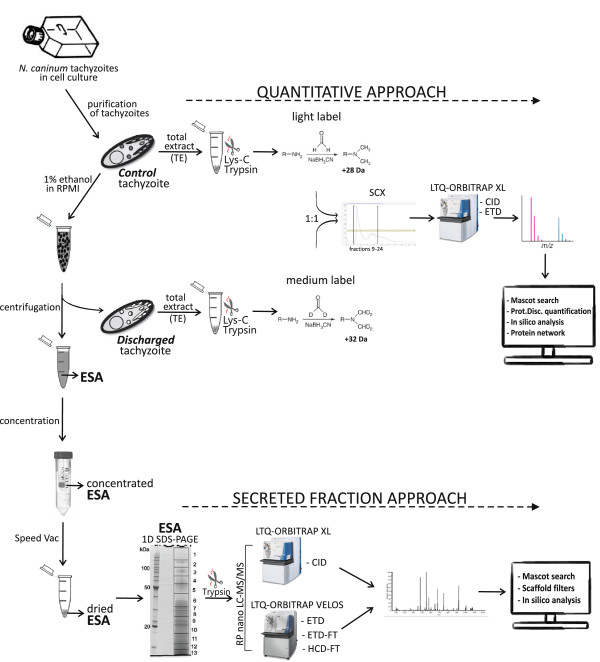
**Experimental design of the *N. caninum *secretome study.** Purified *N. caninum* tachyzoites were stimulated with 1% ethanol, and the ESA proteins were separated from the *discharged* tachyzoites. For the Secreted Fraction Approach, the ESA was concentrated, dried and after separation with 1D SDS-PAGE, the proteins were digested. The tryptic peptides were analysed with the following: an LTQ-Orbitrap-XL, equipped with a peptide fragmentation system by collision induced dissociation (CID), and an LTQ-Orbitrap-Velos, using a decision tree-guided peptide fragmentation, equipped with electron transfer dissociation (ETD) with LTQ mass analysis, ETD with Orbitrap readout (ETD-FT), and higher-energy collisional dissociation (HCD-FT) with Orbitrap readout. For the Quantitative Approach, unstimulated tachyzoites (*control*) and the tachyzoites recovered after ESA collection (*discharged*), separately, had their total protein extract (TE) produced and digested with Lys-C and trypsin. The peptides were dimethyl labelled with light (*control*) and medium (*discharged*) labelling reagents, mixed in a 1:1 ratio, fractionated by strong-cation exchange (SCX) and analysed on an LTQ-Orbitrap-XL using a decision tree for the fragmentation methods CID and ETD. For both approaches, combined MS data were analysed using Mascot software and *in silico* analyses were performed (details in Methods).

The entire experimental design is schematically represented in Figure 1.

### Identification of proteins from the tachyzoite-secreted fraction (ESA)

#### N. caninum tachyzoite culture and purification

*N. caninum* tachyzoites of the Nc-1 isolate were cultured on Vero cell monolayers in RPMI-1640 medium (Sigma-Aldrich) supplemented with 2.05 mM glutamine and 0.1 mg/mL kanamycin at 37°C and 5% CO_2_ in T-25 cm^2^ and T-75 cm^2^ tissue culture flasks.

After partial destruction of the Vero cell monolayers, the *N. caninum* tachyzoites were passaged five times through a 26 G x ½ in. needle to disrupt the cells, filtered through a 5 μm syringe filter and subjected to size-exclusion chromatography in PD10 columns (Sephadex G-25, GE). After purification, the tachyzoites were subjected to flow cytometry (FACSCanto flow cytometer and FACSDiva software, BD) to evaluate their viability through the incorporation of 20 μg/mL propidium iodide. Using heat-killed tachyzoites (85°C, 10 min) as controls, 90% of the tachyzoites were confirmed to be alive after the purification process (data not shown).

#### Obtaining the secreted fraction (ESA) from N. caninum tachyzoites

The ESA fraction was obtained after the freshly purified tachyzoites were incubated (10^8^/mL) in RPMI medium containing 1% ethanol (Merck) at 37°C for 15 min and cooled for 10 min on ice. The supernatant was purified using a two-step centrifugation method, and the aliquots were stored at −20°C. Using centrifugal concentrators (Amicon Ultra Centrifugal Filter Devices 5 K, Millipore), several ESA aliquots were washed at 4°C with 25 mM ammonium bicarbonate, pH 7.4, followed by MilliQ water, concentrated at 4°C in water to 700 μL and dried in a vacuum concentrator.

#### Protein digestion

The ESA was resuspended using sonication (LABSONIC M, Sartorius) in 120 μL of 50 mM ammonium bicarbonate; 25% of this sample (38.5 μg) was loaded into one lane of a 12% 1D SDS-PAGE gel and stained with colloidal coomassie dye G-250 (Gel Code Blue Stain Reagent, Thermo Scientific). The lane containing the separated ESA proteins was cut into 13 bands, which were treated with 6.5 mM dithiothreitol (DTT) for 1 h at 60°C for reduction and 54 mM iodoacetamide for 30 min for alkylation. The proteins were digested overnight with trypsin (Promega) at 37°C. The peptides were extracted with 100% acetonitrile (ACN) and dried in a vacuum concentrator.

#### Mass spectrometry: RP-nanoLC-MS/MS

The data were acquired using two different mass spectrometers. The same online nanoLC configuration was used for all LC-MS experiments. Prior to MS analyses, the peptides were separated with an Agilent 1200 series LC system equipped with a 100 μm × 20 mm, 3 μm, 120 Å Reprosil C18-AQ double frit trapping column (Dr. Maisch, Ammerbuch, Germany) and a 50 μm × 400 mm, 3 μm, 120 Å Reprosil C18-AQ analytical column (Dr. Maisch, Ammerbuch, Germany). Trapping was performed at 5 μL/min for 10 min in solvent A (0.1 M acetic acid in water), 40% solvent B for 30 min, 100% solvent B for 2 min, and solvent A for 15 min. The flow rate was passively split to 100 nL/min during the elution analysis. The nanospray was performed at 1.7 kV using a fused silica capillary that was pulled in-house and coated with gold (o.d. 360 μm; i.d. 20 μm; tip i.d. 10 μm). The tryptic peptides were analysed both with an LTQ-Orbitrap-XL, equipped with a peptide fragmentation system by collision induced dissociation (CID), and with an LTQ-Orbitrap-Velos, using a decision tree-guided peptide fragmentation. The mass spectrometers were used in a data-dependent mode, which automatically switched between MS and MS/MS.

For both mass spectrometers, full-scan MS spectra (from m/z 350 to 1500) were acquired in the Orbitrap with a resolution of 60,000 at m/z 400 after the accumulation of a target value of 500,000 in the linear ion trap. The five most intense ions were selected for fragmentation in the linear ion trap at a normalised collision energy of 35% after the accumulation of a target value of 10,000.

For the LTQ-Orbitrap-Velos, a decision tree-based method was used. The decision tree-guided peptide fragmentation is based on the charge state and the m/z of the precursor peptide, and a decision is made between electron transfer dissociation (ETD) with LTQ mass analysis, ETD with Orbitrap readout (ETD-FT), and higher-energy collisional dissociation (HCD-FT) with Orbitrap readout only. For details, see Frese *et al*., [25].

#### Protein identification

The data were searched against the *N. caninum* predicted protein database (ToxoDB version 6.4, 7344 entries), including a list of common contaminants (http://maxquant.org/contaminants.zip), using Mascot software version 2.3.02 (Matrix Science). The carbamidomethylation of cysteine and the oxidation of methionine were set as fixed and variable modifications, respectively. The database search was performed using Proteome Discoverer 1.3 software (Thermo Fischer Scientific) with a peptide tolerance of 7 ppm and product ion tolerances of 0.6 Da (ion trap readout) and 0.05 Da (Orbitrap readout), allowing for two missed cleavages. The following filters were applied: a Mascot ion score of at least 20 and a position rank of 1 in the Mascot search. The resulting .MGF data were converted to .DAT files and filtered in Proteome Software Scaffold 3. Peptide and protein identifications were accepted if they could be established at greater than 95.0% probability, and protein that contained at least 1 unique peptide, as specified by the Peptide and Protein Prophet algorithm [26,27].

#### In silico prediction of signals for secretion

The amino acid sequences of the identified *N. caninum* ESA proteins were submitted to the SecretomeP 2.0 Server (http://www.cbs.dtu.dk/services/SecretomeP/) for the prediction of non-classical secretion using the “mammalian” search option. Proteins containing signal peptides predicted according to SignalP were also displayed by SecretomeP [28].

#### Classification of identified ESA proteins

The identified ESA proteins were classified according to their parasite localisation and secretion pathways. For uncharacterised proteins, localisation was primarily predicted based on homologues from closely related apicomplexan organisms, searched through the ToxoDB website [29]. ToxoDB is a member of pathogen-databases that are housed under the Eukaryotic Pathogens Database (EuPathDB) Bioinformatics Resource Center (BRC).

Thus, the list of proteins that were putatively localised to micronemes was obtained using the keyword “micronem*” and proteins that were previously identified in *N. caninum* or a homologue but absent from this list were manually inserted into the final list. The search was performed for other protein groups following the same method: “rhoptr*” for proteins from rhoptries, “dense granul*” for dense granule proteins, “surface*” or “SAG*” (surface antigens) or “SRS*” (surface antigens related sequences) for parasite surface proteins, “cycloph*” for cyclophilins or cyclophilin-like proteins, “mitochond*” for mitochondrial proteins, and “nuclear*” or “nucleus” for nuclear proteins. It was not possible to obtain results in *Neospora* using the word “apicoplast*,” thus, we searched the *T. gondii* ME49 database for a list of *N. caninum* homologues. The nomenclature adopted here for the uncharacterised proteins follows Reid *et al.*[30], however, new nomenclature might be eventually accorded when these proteins are characterised.

### Relative quantification of proteins in the discharged tachyzoite

#### Obtainment of control and discharged tachyzoites

The treatment to obtain the *discharged* tachyzoites was the same for the ESA (section Obtaining the secreted fraction (ESA) from *N. caninum* tachyzoites). After the secretion stimulus, the *discharged* tachyzoites were harvested by centrifugation and stored at −20°C. The control was composed of purified and unstimulated parasites (*control* tachyzoites).

#### Protein extraction from control and discharged tachyzoites

Total protein extracts (TE) from *N. caninum control* and *discharged* tachyzoites were obtained by sonication (Sonic Dismembrator 100 - Fisher Scientific) of approximately 8.5×10^8^ parasites in 200 μL of lysis buffer containing 7 M urea, 2 M thiourea and 4% CHAPS (Sigma-Aldrich), yielding 1 mg of proteins each. The proteins were precipitated in 30% trichloroacetic acid (Sigma-Aldrich) in acetone (Merck) and dried in a vacuum concentrator.

#### Protein digestion and peptide labelling

Each extract was resuspended, with sonication (LABSONIC M - Sartorius), in 300 μL of 8 M urea containing Complete EDTA-free Protease Inhibitor Cocktail (Roche), reduced with 0.4 mM DTT for 25 min at 56°C, and alkylated with 8 mM iodoacetamide for 30 min at room temperature, in the dark. The proteins were first digested with Lys-C (1:75 ratio w/w) at 37°C for 4 h and, after 8 times sample dilution in 50 mM ammonium bicarbonate, digested with trypsin (1:100 ratio w/w) at 37°C, overnight.

Stable isotope dimethyl labelling of peptides was performed as described in the standard protocol [31]. Equal amounts of the extracted peptides from *control* and *discharged* samples were separately labelled with light and intermediate labels, respectively, using an in-solution labelling method and SepPak C18 cartridges (Waters). The percentage of labelled peptides and the correct proportion of labelled *control* and *discharged* samples were checked by mass spectrometry (MS) in a LTQ-Orbitrap-XL, using decision tree guided fragmentation for CID or ETD, with a 60 min running for proportion checking and a 45 min running for labelling checking. *Control* and *discharged* labelled samples were then mixed in a 1:1 ratio.

#### Strong cation exchange fractionation

Strong cation exchange (SCX) fractionation was performed as described previously [32] for the fractionation of the 1:1 mixture containing labelled peptides. SCX was performed using a Zorbax BioSCX-Series II column (0.8 mm inner diameter, 50 mm length, 3.5 μm). Solvents consisted of 0.05% formic acid in 20% acetonitrile (solvent A) and 0.05% formic acid, 0.5 M NaCl in 20% acetonitrile (solvent B), used in the followed gradient: 0–0.01 min (0–2% B); 0.001–8.01 min (2–3% B); 8.01–18.01 min (3–8% B); 18.01–28 min (8–20% B); 28–38 min (20–40% B); 38–44 min (40–100% B); 44–48 min (100% B); and 48–50 min (100–0% B), at a flow rate of 40 μL/min. Between 40 and 90 minutes, 50 fractions of 1 minute were collected.

#### Mass spectrometry (MS)

For MS analysis, the 15 most intense fractions, containing doubly and triply charged peptides, were selected and reconstituted in 10% formic acid and 5% DMSO. Prior to MS, peptides were separated using the same online nanoLC configuration as described in section Mass spectrometry: RP-nanoLC-MS/MS. The analysis of the peptides was performed on an LTQ-Orbitrap XL mass spectrometer (section Mass spectrometry: RP-nanoLC-MS/MS), equipped with a decision tree-based method for peptide fragmentation, in which a decision is made between collision induced dissociation (CID) and electron transfer dissociation (ETD) based on mass and charge, as described elsewhere [25]. Trapping was performed at a flow of 5 μL/min for 10 min, and the fractions were eluted using a 3 h gradient from 0 to 40% solvent B [0.1 m acetic acid in 80% ACN (v/v)] at 100 nL/min. The 10 most intense ions were selected for fragmentation in the linear ion trap at a normalised collision energy of 35% after accumulation of a target value of 10,000.

#### Data analysis

The raw data was analysed with the Proteome Discoverer 1.3 software (Thermo Fischer Scientific). The MS/MS spectra were searched against the *N. caninum* predicted protein database (version 6.4), including a list of common contaminants (section Protein identification) and a decoy database (composed of the reversed versions of all sequences), with Mascot software version 2.3.02 (Matrix Science). Trypsin was set for enzyme specificity allowing for two missed cleavages. Carbamidomethylation (C) and oxidation (M) were set as fixed and variable modifications, respectively. Additionally, due to the dimethyl labelling, dimethyl (K), dimethyl (N-term), dimethyl-2H(4) (K) and dimethyl-2H(4) (N-term) were set as variable modifications. Using a peptide tolerance of 5 ppm and product ion tolerance of 0.6 Da, and applying the peptide filters of position rank of 1, Mascot ion score higher than 20, and peptide length between 7 and 35 residues, the resulting false discovery rate (FDR) was 1.2%.

Quantification was carried out with Proteome Discoverer 1.3 software (Thermo Fischer Scientific) using peak area with the ‘precursor ions quantifier’ node. Only unique peptides were quantified and the ratio reporting was set as medium/light, where medium labeled peptide intensities (peak areas) from the *discharged* sample were divided by the light labeled peptide intensities from the *control* sample. Ratios were normalised on the protein median and a fold change threshold of 2.0 was set for up or down regulation of proteins in the *discharged* tachyzoite.

#### Classification by protein localization

The quantified proteins were classified as described in section *In silico* prediction of signals for secretion according to their parasite localisation (micronemes, rhoptries, dense granules and surface).

### Interaction network

The prediction of protein-protein interactions among the 2,011 quantified proteins was performed by using the Universal Predictor of Protein-Protein Interactions (UNISPPI) [33]. UNISPPI is a machine learning-based computational method that predicts the probability of physical interaction between any pair of proteins, based solely on their physicochemical features associated with amino acid sequences. The physicochemical features of the identified proteins’ amino acid sequences were calculated with the PROFEAT software [34]. The interacting protein pairs, with probability higher than 90%, containing up or down regulated proteins were selected for their visualisation with the software Cytoscape version 2.8 [35], using the Cytoscape layout Edge-Weighted Force-Directed (BioLayout) using as weight the edge betweenness, a network centrality that is defined as the number of the shortest paths that go through an edge in the network. The networks of up and down regulated proteins were combined to build a net union containing the interactions from both networks.

## Results and discussion

Our study to comprehend the *N. caninum* secretome was based on the secretory stimulus via increased intracellular Ca^2+^, *in vitro* triggered by ethanol. The implementation of two different but connected approaches, employing techniques that ensure high resolution and sensitivity, enabled the identification of several secreted proteins and allowed for the search of new invasion-related targets.

### Proteins identified from the *N. caninum* secreted fraction (ESA)

The collected ESA proteins were separated into 13 bands by 1D-SDS-PAGE (Figure 1) and were in-gel digested. The tryptic peptides were analysed by nanoLC-MS/MS, resulting in a total of 4,941 identified peptides, corresponding to a total of 615 proteins, after the appropriate cut-off filters were applied to the results. The Additional file 1: Table AF1 (sheet A) lists all the proteins identified displayed according to the number of protein abundances, obtained after dividing their spectral counts by their molecular weights, to prevent biases caused by protein size [36]. The excel files containing information about the peptides’ sequence and charge state used to identify each protein are compressed and available in Additional file 2 (615_ESA_proteins.rar). Proteins predicted to be from the secretory organelles (micronemes, rhoptries, and dense granules) or parasite surface, are displayed in Additional file 1: Table AF1 (sheet B), arranged in a descending order of relative abundances in ESA.

**Figure 2:**
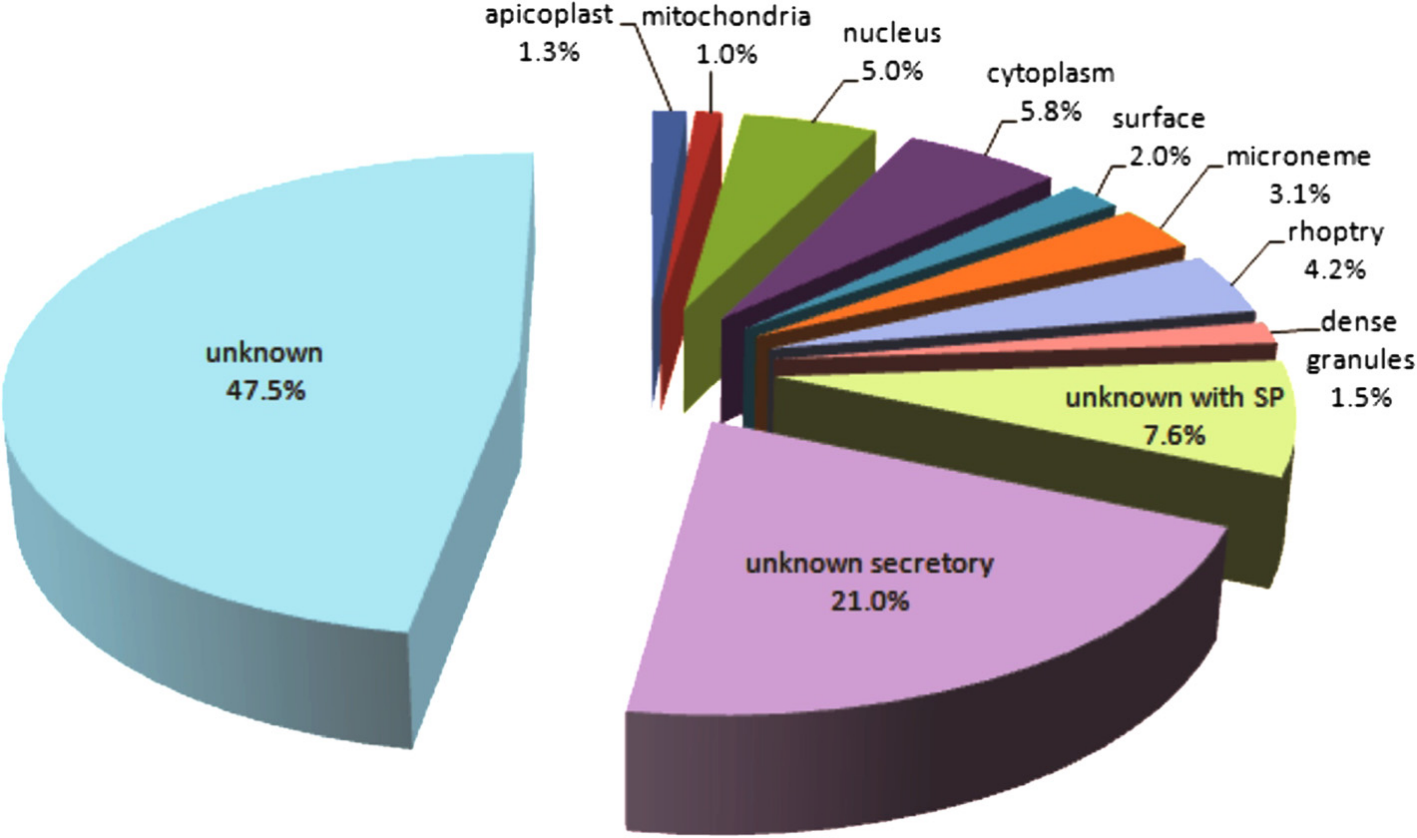
**Percentage of proteins in the ESA of *N. caninum *tachyzoites, from a total of 615 identified proteins.** Classification was based on parasite localisation (confirmed or putative) and predicted secretion pathways (classic secretion, unknown with signal peptide (SP); non-classical secretion, unknown secretory (predicted by Secretome P). Unknown, unknown localisation.

Of the 615 ESA proteins identified (Figure 2), 37.4% (n = 230) corresponded to secreted or putatively secreted proteins: 3.1% (n = 19) were microneme proteins, 4.2% (n = 26) rhoptry proteins, 1.5% (n = 9) dense granule proteins, 7.6% (n = 47) proteins with unknown localisation but containing signal peptides (SP), and 21% (n = 129) were proteins predicted to have non-classical secretion (“unknown secretory”). The high percentage of proteins predicted to be secreted via non-classical pathways corroborates studies on other organisms, in which some alternative routes were associated with secretion via vesicles [37,38]. Proteins from the parasite surface, which can accumulate in the ESA as a result of proteolytic shedding from the parasite [39], represented 2.0% (n = 12) of the proteins. Proteins from non-secretory organelles also appeared in the *N. caninum* ESA: eight proteins from the apicoplast (1.3%), six from the mitochondria (1.0%), 36 from the cytoplasm (5.8%), and 31 from the nucleus (5.0%). These proteins not expected to be secreted may have been released from dead tachyzoites, since flow cytometry showed that approximately 10% of the tachyzoites were dead after purification.

Proteins with unknown localisations and without secretory signals totalled 47.5%. Additional file 1: Table AF1 (sheet C) lists proteins from the apicoplast, mitochondria, cytoplasm and nucleus. Another interesting group of proteins found in our results, included as “unknown” in Figure 2, contained eight cyclophilins (1.3%, Additional file 1: Table AF1 sheet B); of which only one (18 kDa cyclophilin) had been previously identified and characterised [40]. In *N. caninum* and *T. gondii*, 18 kDa cyclophilin displays monocyte chemoattractant properties, as it mimics the ligands of CC-chemokine receptor 5 (CCR5), inducing cell migration to infection sites and enhancing parasite invasion and proliferation [41,42].The other seven identifications refer to putative cyclophilins or cyclophilin-like proteins and, like in other Apicomplexan organisms [43], they are composed of the Cyclophilin (Cyp) domain (NCLIV_019970, NCLIV_017250, NCLIV_070210); the Cyp combined to a signal peptide (NCLIV_036200); or the Cyp combined to other domains, such as RNA recognition motif - RRM (NCLIV_045550), WD40 repeat (NCLIV_009630), and FK506-binding domain (FKBP) plus tetratricopeptide repeat (TPR) (NCLIV_028870). Thus, the presence of many cyclophilins in the *N. caninum* ESA may regulate RNA processing, host cell migration and parasite proliferation [41-43].

### Relative quantification of proteins in the *discharged* tachyzoite

#### Identification and relative quantification of proteins

The quantitative approach was performed to avoid the contamination of proteins from dead tachyzoites and to observe secreted proteins among the “down-regulated” group of proteins.

The peptides from *discharged* and *control* samples were labelled with stable isotopes using dimethyl triplex labelling, allowing robust and accurate relative quantification [31,44]. The labelled samples were mixed and fractionated using strong cation exchange (SCX) and the 15 most abundant fractions were selected for MS analysis. From a total of 2,163 identified proteins, 2,011 were quantified. Additional file 3: Table AF3 displays all the identified and quantified proteins, disposed according to their medium/light (*discharged/control*) ratio, and information about identification and quantification of proteins and peptides.

For further analysis we chose a fold-change threshold of 2 for the *discharged/control* ratio, resulting in 150 proteins being up-regulated (log_2_ ratio higher than 1) and 90 proteins down-regulated (log_2_ ratio lower than −1) in the *discharged* tachyzoite. Additional file 4: Table AF4 lists the up (sheet A) and down (sheet B) regulated proteins.

**Figure 3:**
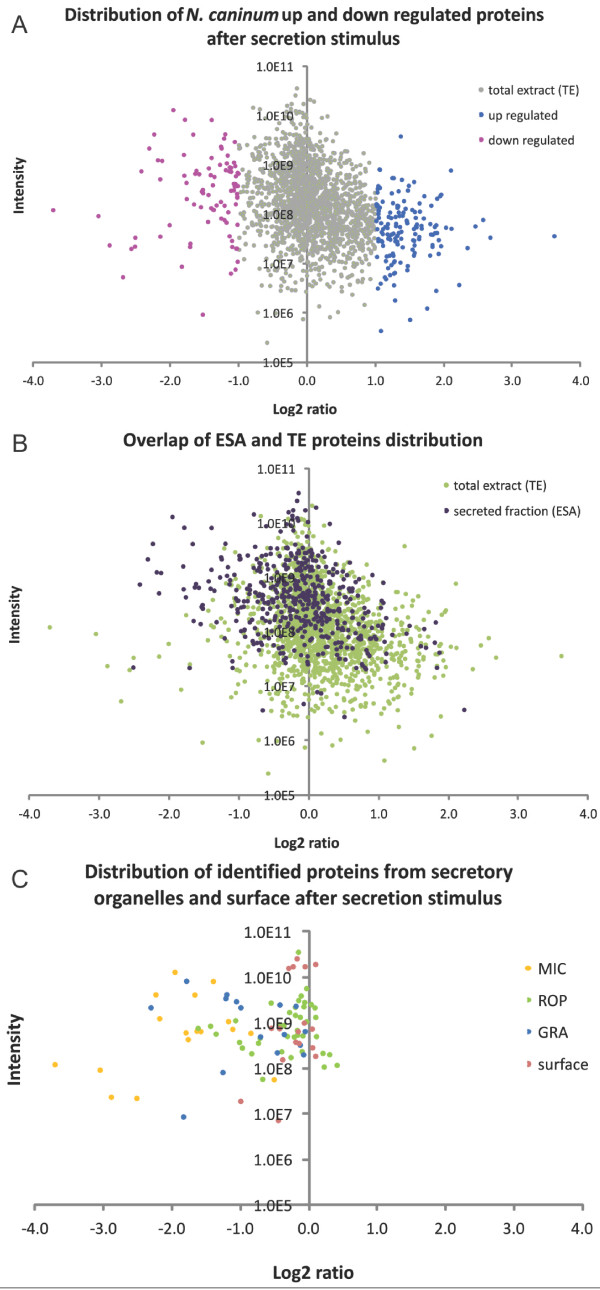
**Distribution of the *N. caninum *quantified proteins after secretion stimulus. A**, Distribution of the proteins in the *discharged* tachyzoite (ratio *discharged/control*). Up-regulated proteins are in blue and down-regulated proteins are in pink. **B**, Comparison of the distribution of proteins identified from *N. caninum* secreted fraction (ESA) to the proteins from the total extracts (TE). ESA proteins are in purple, TE proteins are in light green. **C**, Ratio distribution of the proteins identified in the TE, from secretory organelles. MIC, micronemes (yellow); ROP, rhoptries (green); GRA, dense granules (blue); surface (light pink).

The distribution of the quantified proteins from *N. caninum* total extract (TE) was plotted for visualization of the protein log_2_ ratios against their intensities. The up-regulated proteins in the *discharged* tachyzoite are highlighted in blue, while the down-regulated are highlighted in pink (Figure 3A). Figure 3B depicts the proteins identified in both ESA and TE (n = 497) coloured in purple in the ESA protein distribution. Finally, Figure 3C depicts the proteins localised to the parasite surface and to the organelles micronemes, rhoptries and dense granules among the quantified proteins and their ratios plotted against their intensities. Additional file 5: Table AF5 (sheet A) displays the proteins found in each group with their *discharged/control* ratio*.*

De facto, microneme proteins, which were experimentally shown to be secreted in response to Ca^2+^ increase [7], represented a great percentage of the down-regulated proteins (16.7%), and the sum of microneme, rhoptry and dense granule proteins corresponded to 30.1% of the down-regulated proteins. Overlaying the proteins identified in ESA and quantified by dimethyl labelling with all dimethyl-quantified proteins (Figure 3B) demonstrated a preference to the left side of the log_2_ scale, meaning down regulation of these specific proteins. While most of the microneme proteins have a log_2_ ratio of less than −1 (Figure 3C), most of the rhoptry and dense granule proteins show ratios higher than micronemes, albeit still negative.

### Comparison of both approaches

**Figure 4:**
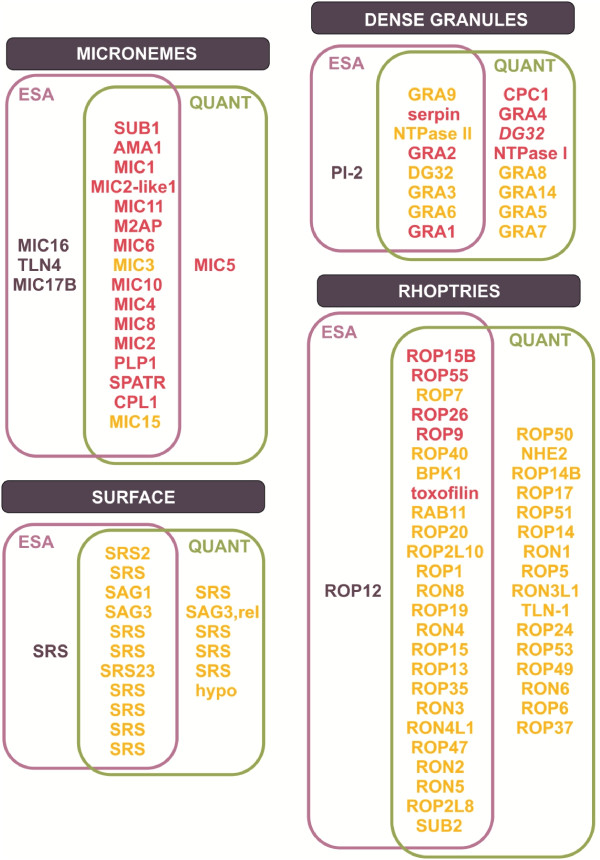
**Schematic representation of identified proteins between both approaches: secreted fraction (ESA) and relative dimethyl quantification (QUANT).***N. caninum* proteins identified in the present study were grouped according to their known or putative localisation. Proteins in the purple zone were identified by the ESA approach; proteins in the green zone were identified by the quantitative approach; proteins in the intersection of purple and green zones were identified by both approaches. ESA proteins are arranged in descending order of protein abundance. QUANT proteins in red belong to the down-regulated group, and in yellow were classified as not differentially expressed.

Both approaches were compared (Figure 4, details in Additional file 5: Table AF5. B). It is notable that several highly abundant proteins identified in ESA, such as MIC3, GRA9 and ROP7, although identified in the dimethyl labelling experiment, did not appear as being down regulated. This could indicate that the fold change threshold of 2 might be too strict, since the mechanism of protein release from the three organelles is still not completely understood. However, the comparison between the ESA and TE was advantageous to more precisely determine the most likely secreted proteins.

A total of 20 microneme proteins were identified (Figure 4), wherein three were identified only in ESA (MIC16, TLN4 and MIC17B) and one only in TE (MIC5). The three proteins solely from ESA, although not so abundant, may take part in parasite invasion since MIC16 is a new transmembrane protein containing putative TSR (thrombospondin) type 1 domains and is susceptible to rhomboid cleavage [45]; MIC17B contains adhesive Apple domains and was identified from a *N. caninum* organelle fraction by monoclonal antibody [21], and toxolysin 4 (TLN4) is a putative metalloproteinase processed into multiple proteolytic fragments within the parasite secretory system, and some of these proteolytic fragments remain associated in a large molecular complex [46]. MIC5, in *T. gondii*, occupies the parasite surface during invasion and regulates the adhesive activity of other secreted proteins [47]. Highly abundant and identified by both approaches, SUB1 [48], AMA1 [49], and MIC1 [50] are extensively studied microneme proteins. Other *N. caninum* microneme proteins, such as MIC2-like1 [51], MIC3 [52], MIC10 [53], MIC4 [54] and MIC2 [55], were also highly abundant in the ESA. M2AP, MIC6, and MIC8 are well-characterised microneme proteins in other apicomplexan parasites [56-58]; in contrast to *N. caninum*, where identification was only from genomic or gene expression profile studies [30,59]. One of the most abundant *N. caninum* ESA proteins, MIC11, has been shown to stimulate bovine CD4 + naive T cells [22]. Less abundant proteins, such as PLP1, SPATR, CPL1 and MIC15, were identified in *N. caninum* for the first time, and play important roles in other apicomplexan parasites. In *T. gondii*, PLP1, a perforin-like protein secreted from micronemes, aids in the parasite egress from the parasitophorous vacuole membrane [60]. SPATR [61], CLP1 and MIC15 [Carruthers 2010 – unpublished observations from ToxoDB] are proteins with cell invasion-related domains.

The signalling pathways for rhoptries and dense granules secretion are not elucidated. It is supposed that, following receptor-engagement by microneme proteins, their cytoplasmic domains may play a role in signal transduction leading to rhoptry release [62]. Interestingly, several proteins from rhoptries and dense granules were identified from *T. gondii* sporocyst/sporozoite fractions [63]. However, two proteomic *T. gondii* studies using tachyzoites had poorly identified proteins from rhoptries and dense granules [7,39]. In contrast, we identified a large number of proteins from these two organelles, probably due to increased sensitivity.

Numerous rhoptry proteins were detected (n = 42), the majority in both ESA and TE (n = 25), one solely in ESA, and 16 proteins only in TE. Many of these proteins, including abundant ones, such as ROP15B, ROP26 and toxofilin, have been previously detected only in genome or transcriptome *N. caninum* studies [30]. The BPK1 and ROP15 genes had their expression levels reduced in a temperature-sensitive *N. caninum* mutant (relatively avirulent), suggesting their roles in parasite virulence [59]. RON2, RON4, RON5, and RON8 are rhoptry neck proteins that comprise the moving junction complex AMA1-RON2/4/5/8 in *T. gondii*[64]. In a *N. caninum* lysate, RON2 and RON5 reacted with *T. gondii* antiserum, and coimmunoprecipitation indicated them as part of the *N. caninum* moving junction complex, together with RON4 and RON8 [65]. Some proteins were predicted to be rhoptry kinases, as the paralogs ROP19 and ROP47, and some may act as proteases, like TLN1 and SUB2, which were characterised in *T. gondii*[66,67].

Although less abundant than microneme or rhoptry proteins, some interesting dense granule proteins were found; eight were identified by both approaches, one only in ESA, and eight solely in TE. The most abundant dense granule proteins in ESA were GRA9, serpin (NCLIV_063150), NTPaseII, and GRA2. Two members of the serpin family (serine protease inhibitors) were identified; protease inhibitor 2 (PI-2) demonstrated to have activity against trypsin in *T. gondii*[68], and serpin NCLIV_063150, most likely a new member of the serpin family, as it is closer to an undescribed putative serpin of *T. gondii* (TGME49_246130). TgPI-1 is secreted from dense granules into the parasitophorous vacuole (PV) and inhibits trypsin, chymotrypsin, and neutrophil elastase inhibitor, suggesting a possible protection role for the parasite [69]. The presence of these serine proteases might be important for the ability of the parasite to survive within its host [68].

Twelve surface proteins were identified in ESA and, although 11 were also quantified, none belonged to the down-regulated group. Six other putative surface proteins were detected only in TE. SRS2, a potential vaccine candidate for neosporosis [70,71], was highly abundant in the *N. caninum* ESA, as well as SAG1 and SAG3, which are vaccine targets against toxoplasmosis [72-74]. Three proteins (NCLIV_010720, NCLIV_010730 and NCLIV_068920) compose a group of four SRS protein paralogs in *N. caninum*. Another group of SRS protein homologues, including NCLIV_068872 and NCLIV_052740, contains 15 paralogs that do not exist in *T. gondii*.

### Proteins putatively involved in secretion signalling pathways

Proteins putatively involved in the secretion process were also investigated in the present work. *Plasmodium falciparum* and *T. gondii* studies indicate that, upon contact with the host cell, the second messengers inositol triphosphate (IP3) and cyclic ADP ribose (cADPR) are generated and bind to calcium channels putatively localised in the ER, releasing the stored Ca^2+^[9,75]. Ca^2+^ mobilisation signals, such as the activation of CDPKs (calcium dependent protein kinases) and the activation of kinases by cAMP and cGMP, regulate the exocytosis of invasion organelles and the activation of the invasion motor [9,62,75]. Three metabolic pathways sensitive to Ca^2+^ increase are, according to the Library of Apicomplexan Metabolic Pathways (LAMP) [76], the inositol phosphate, purine, and pyrimidine. The proteins from these pathways are highlighted in Additional file 6: Figure S1 and Additional file 7: Figure S2 according to their quantification in this work (Additional file 8: Table AF8, sheets A, B and C). Thirteen proteins were related to the inositol phosphate metabolism, where phosphoinositide 5-phosphate (NCLIV_000140) presented down regulation, which facilitates the accumulation of the IP3 precursors (Additional file 6: Figure S1). Five homologues of the enzyme phosphatidylinositol-4-phosphate-5-kinase, which favours the synthesis of the IP3 precursors, were identified. For the purine metabolism 25 proteins were identified. It is notorious that the synthesis of inosine monophosphate (IMP) is favoured, since adenosine kinase, which consumes adenosine, was down-regulated, while AMP deaminase (AMP → IMP) was up-regulated. The accumulated IMP may result in an increase of GDP (precursor of cGMP), since the enzymes GMP synthase and guanylate kinase were up-regulated (Additional file 7: Figure S2).

Other proteins related to increased Ca^2+^ levels were identified by the quantitative approach, such as the homologues of *T. gondii* phosphoproteins (Additional file 8: Table AF8, sheet D), potentially involved in mediating intracellular signalling cascades, regulating exocytosis of invasion organelles, or controlling parasite motility [75]. Nebl and colleagues have used the same ethanol stimulus and quantitative analyses of the stimulated parasite to reveal calcium-dependent phosphorylation sites. As an example found in both studies, the armadillo repeat-containing protein (ARM1) was up-regulated in our study and, although not yet characterised, animal proteins containing ARM repeats function in important processes, including intracellular signalling and cytoskeletal regulation [77]. The core components of the invasion motor MyoA, MLC1, GAP40, GAP45, GAP50 and ELC1 were here also identified; however, even though motility appears to be dependent on calcium signalling transduction [75], none were up-regulated. Calcium-dependent protein kinases (CDPKs), implicated in mediating crucial calcium-dependent signal transduction pathways in apicomplexan parasites [9] were also found. In *T. gondii*, CDPK1 is an essential regulator of calcium-dependent micronemal exocytosis [78]; but no syntenic homologue is predicted in *N. caninum*. The highest expressed CDPK in our study was CDPK2A, followed by CDPK7. Furthermore, other cell-cycle-associated kinases (CMGC), such as NCLIV_020950, NCLIV_007880 and NCLIV_001240, were up regulated (Additional file 8: Table AF8, sheet D).

### Interaction network

The exploration of protein-protein interactions is a new strategy to identify antimalarial drug targets, which should have effects on important functional nodes controlling crucial networks for parasite survival [79]. The physical interactions among the 2,011 quantified proteins were predicted with the Universal Predictor of Protein-Protein Interactions (UNISPPI) [33]. Additional file 9: Table AF9 displays all predicted interactions with probabilities higher than 90% (sheet A); and the pairs of interacting proteins involving the up- or down-regulated proteins (sheet B).

**Figure 5:**
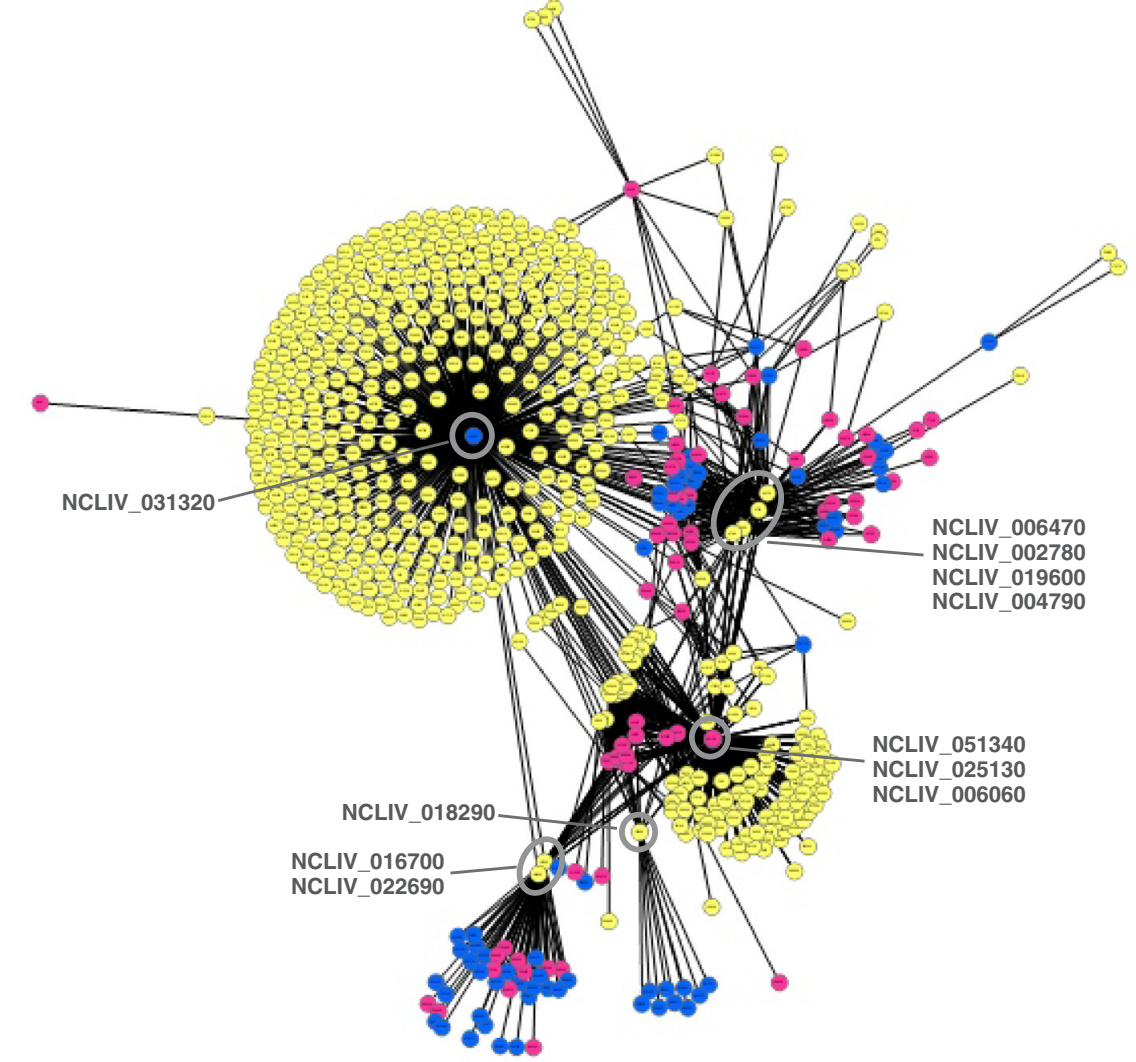
**Cytoscape visualisation of interaction network involving up and down regulated proteins in *N. caninum discharged *tachyzoite.** The *discharged*/*control* tachyzoite ratios defined the protein classification in up-regulated (log_2_ ratio higher than +1.0; nodes in blue), down-regulated (log_2_ ratio lower than −1.0; nodes in pink), or non-differentially expressed proteins (log_2_ ratio between −1.0 and +1.0; nodes in yellow). NCLIV_031320 is an up-regulated protein predicted to interact with 466 proteins; NCLIV_051340, NCLIV_025130 and NCLIV_006060 are down-regulated proteins showing 129 predicted interactions; NCLIV_018290, NCLIV_016700, NCLIV_022690, NCLIV_006470, NCLIV_002780, NCLIV_019600 and NCLIV_004790 are proteins predicted to interact with both up and down regulated proteins.

The interaction network involving up and down regulated proteins (Figure 5, details in Additional file 10: Figure S3) revealed central proteins involved in the calcium-induced pathways and potentially in invasion. The up-regulated protein NCLIV_031320 exhibited the highest number of interactions (466 proteins). NCLIV_031320 is a potential RNA binding protein, since it contains a nuclear localisation signal (NLS) [80,81] plus a Gly-rich region, prone to promote homo and heteromeric interactions to create ribonucleoprotein (RNP) complexes [82]. Three down-regulated proteins presented 129 interactions: toxofilin (NCLIV_051340); a putative translation initiation factor 1 (NCLIV_025130); and a hypothetical protein (NCLIV_006060). Other non-differentially expressed proteins showed a high number of interactions: the homologue of *T. gondii* small GTPase Rab6 (NCLIV_054540) with 168 interactions, and a putative receptor for activated C kinase RACK (NCLIV_059430) with 148 interactions. Interestingly, six proteins were predicted to interact both with up- and down-regulated proteins, ergo strong candidates to be involved in the invasion process (Additional file 9: Table AF9 C). From these six proteins, only one has been characterised, the 18 kDa cyclophilin (NCLIV_004790) [40].

## Conclusion

In summary, this secretome study explored two different approaches using high-resolution nanoLC-MS/MS. The comparison between the protein abundances in ESA and their measure in the *discharged* tachyzoite allowed for a more precise identification of the most likely secreted proteins. Information from the network interaction and up-regulated proteins was important to recognise key proteins potentially involved with the metabolic regulation of secretion. Our results may be helpful to guide the selection of targets to be investigated for therapy against *Neospora caninum* and other Apicomplexan organisms.

### Associated content

The mass spectrometry proteomics data have been deposited in the ProteomeXchange Consortium (http://proteomecentral.proteomexchange.org) via the PRIDE partner repository [83] with the dataset identifier PXD000424.

## Abbreviations

1D: One dimension; ACN: Acetonitrile; AMA1: Apical membrane antigen 1; cAMP: Cyclic adenosine monophosphate; cGMP: Cyclic guanosine monophosphate; CID: Collision-induced dissociation; CCR5: CC-chemokine receptor 5; CPC: Cathepsin C; CyP: Cyclophilin; dCDP: Deoxycytidine diphosphate; dUDP: Deoxyuridine diphosphate; DG: Dense granule; DTT: Dithiothreitol; ESA: Tachyzoite secreted fraction; ETD: Electron transfer dissociation; FDR: False discovery rate; FT: Fourier transform ion cyclotron resonance; GRA: Dense granule protein; HCD: Higher-energy collisional dissociation; IMP: Inosine monophosphate; LC: Liquid chromatography; LTQ: Linear trap quadrupole; M2AP: MIC2-associated protein; MGF: Mascot generic format; MIC: Microneme protein; MS: Mass spectrometry; MS/MS: Tandem mass spectrometry; NTPase: Nucleoside triphosphate; ppm: Parts per million; PV: Parasitophorous vacuole; RON: Rhoptry neck protein; ROP: Rhoptry protein; RP: Reversed phase; SAG: Surface antigens; SCX: Strong-cation exchange; SDS-PAGE: Sodium dodecyl sulfate - polyacrylamide gel electrophoresis; SP: Signal peptide; SRS: Surface antigen-related sequences; SUB: Subtilisin-like protease; TE: Total protein extracts; TM: Transmembrane; TLN: Toxolysin.

## Competing interests

The authors declare that they have no competing interests.

## Authors’ contributions

LPO carried out the experiments and drafted the manuscript. HP helped carrying out various aspects of the M/S experiments. MLA and NL helped with the interaction network prediction experiments and revised the manuscript. HvdT and VT helped with the development of bioinformatic tools and revised the manuscript. AJRH, AFM and APY conceived and designed the study and made critical revisions to the manuscript. All authors have read and approved the final manuscript.

## Supplementary Material

Additional file 1 **Table AF1 Proteins identified in the ESA from *N. caninum *tachyzoites. A.** All 615 proteins indentified in the ESA from *Neospora caninum* tachyzoites, discriminated by their ToxoDB ID and arranged in descending order of relative abundance. Information is given on predicted molecular weight, protein probabilities, number of spectrum counts, relative protein abundance (spectrum counts divided by molecular weight), number of unique spectrum counts, number of unique peptides, and percentage of total spectra. The consulted high throughput studies are displayed below the table. The files containing information about the peptide sequences and charge state used to identify each protein are compressed and available in Additional file 2 (615_ESA_proteins.rar). **B.** ESA proteins from *Neospora caninum* tachyzoites displayed by their known or predicted protein groups: micronemes, rhoptries, dense granules, surface and cyclophilins. Proteins are arranged in descending order of relative abundance, and the references of protein identification and/or characterisation are included. **C.** ESA proteins from *Neospora caninum* tachyzoites displayed by their known or predicted protein groups: apicoplast, mitochondria, cytoplasm and nucleus. Proteins are arranged in descending order of relative abundance, and the references of protein identification are included. The consulted high throughput studies are displayed below the table. Click here for file

Additional file 2 **Peptides used in the identification of ESA proteins.** In each file, information is given on peptide sequence and charge state of all peptides used to identify each of the 615 proteins present in the ESA from the Neospora caninum tachyzoite. Click here for file

Additional file 3 **Table AF3 Proteins from *N. caninum *Quantitative Approach. A.** Proteins identified and quantified in the total extract of the *N. caninum discharged* tachyzoite, decreasingly disposed according to their medium/light (*discharged/control*) ratio. Information is given on protein coverage; number of proteins, unique peptides, peptides, and peptide spectrum matches (PSMs); peak area; and medium/light ratio. The number of amino acids, predicted protein molecular weight (MW), and predicted isoeletric point (pI) are displayed in the last columns. Information of each fragmentation method (CID or ETD) on protein score, coverage, number of peptides and PSMs are also included. The sequences of the identified peptides can be visualised by clicking the “+”. **B.** Proteins identified and quantified in the total extract of the *N. caninum discharged* tachyzoite. The medium/light (*discharged/control*) ratios and the respective log_2_ ratios are displayed. Click here for file

Additional file 4 **Table AF4 Up- and down-regulated proteins in the discharged *N. caninum *tachyzoite. A.** Proteins in the UP-REGULATED group of the *N. caninum discharged* tachyzoite, arranged in descending order of log_2_ ratios (*discharged/control*). Information is given on their peak area; known or predicted localisation, or their protein group (when available); signal peptide (SP), transmembrane domains (TM); presence of these proteins in the *N. caninum* ESA; gene ontology; and protein domains. **B.** Proteins in the DOWN-REGULATED group of the *N. caninum discharged* tachyzoite, arranged in descending order of log_2_ ratios (*discharged/control*). Information is given on their peak area; known or predicted localisation, or their protein group (when available); signal peptide (SP), transmembrane domains (TM); presence of these proteins in the *N. caninum* ESA; gene ontology; and protein domains. Click here for file

Additional file 5 **Table AF5 Quantified proteins classified by localisation. A.***N. caninum* quantified proteins classified by known or predicted localisation (microneme, rhoptry, dense granules or surface). *ratio < 0.5, down-regulated (pink); 0.5 < ratio < 2.0, not differentially expressed (yellow); > 2.0, up-regulated (no example present in this table). **B.** Comparison of the proteins identified from the ESA with the ones from the total extracts (TE). Proteins are classified by known or predicted localisation (microneme, rhoptry, dense granules or surface). *, identified only in ESA. **, identified only in TE. *ratio < 0.5, down-regulated (pink); 0.5 < ratio < 2.0, not differentially expressed (yellow); > 2.0, up-regulated (no example present in this table). Click here for file

Additional file 6 **Figure S1 Inositol phosphate metabolism adapted from LAMP (Library of Apicomplexan Metabolic Pathways).** The quantified proteins in *N. caninum discharged* tachyzoite are surrounded by coloured circles designating their expression level (blue, up-regulated; pink, down-regulated; yellow, not differentially expressed), and their log_2_ ratios are also displayed. Click here for file

Additional file 7 **Figure S2 Purine metabolism adapted from LAMP (Library of Apicomplexan Metabolic Pathways).** The quantified proteins in *N. caninum discharged* tachyzoite are surrounded by coloured circles designating their expression level (blue, up-regulated; pink, down-regulated; yellow, not differentially expressed), and their log_2_ ratios are also displayed. Click here for file

Additional file 8 **Table AF8 Proteins related to pathways linked to calcium mobilisation. A.** Proteins quantified in the *discharged* tachyzoite that are involved in inositol phosphate metabolism, searched in Library of Apicomplexan Metabolic Pathways - LAMP [76]. Proteins are sorted by their EC numbers (Enzyme Commission). The protein intensities (peak area) and ratios (medium/light or *discharged/control*) are displayed. **B.** Proteins quantified in the *discharged* tachyzoite that are involved in the purine metabolism, searched in Library of Apicomplexan Metabolic Pathways - LAMP [76]. Proteins are sorted by their EC numbers (Enzyme Commission). The protein intensities (peak area) and M/L ratios (medium/light or *discharged/control*) are displayed. The ratio values lower then 0.5 (or close) are shown in pink and represent down-regulated proteins. The ratio values higher then 2.0 (or close) are shown in blue and represent up-regulated proteins. **C.** Proteins quantified in the *discharged* tachyzoite that are involved in the pyrimidine metabolism, searched in Library of Apicomplexan Metabolic Pathways - LAMP [76]. Proteins are sorted by their EC numbers (Enzyme Commission). The protein intensities (peak area) and M/L ratios (medium/light or *discharged/control*) are displayed. The ratio values lower then 0.5 (or close) are shown in pink and represent down-regulated proteins. The ratio values higher then 2.0 (or close) are shown in blue and represent up-regulated proteins. **D.** Proteins quantified in the *discharged* tachyzoite putatively involved in invasion. Proteins were classified as components of the invasion motor; proteins involved in signalling cascades, organelle exocytosis, or parasite motility; and kinases. M/L ratios (medium/light or *discharged/control*) are displayed. The ratio values lower then 0.5 (or close) are shown in pink and represent down-regulated proteins. The ratio values higher then 2.0 (or close) are shown in blue and represent up-regulated proteins. The *T. gondii* homologues previously identified in Nebl *et al.* 2011 [75] are included. Click here for file

Additional file 9 **Table AF9 *N. caninum *interaction network. A.** Predicted protein interactions among the 2,011 quantified *N. caninum* proteins, with probability higher than 90% (0.9). The protein IDs are the ToxoDB accession numbers without the “NCLIV_” and zeros at the left side of the numbers (for example, NCLIV_053220 is shown as 53220). **B.** Predicted protein interactions involving up-regulated or down-regulated proteins in *N. caninum*. Networks involving up-regulated (blue) or down-regulated (pink) proteins. The pairs of interacting proteins (protein_1 and protein_2) are displayed. In the next columns, the ID of the up- or down-regulated component in the protein pair is evidenced, and its log_2_ ratio is provided. All the up- (blue) and down- (pink) regulated proteins involved in the interaction networks are listed in columns E and K, respectively. **C.** Additional interacting proteins predicted among the 2,011 quantified proteins. The proteins with the highest numbers of interactions are displayed. They are sorted according to their classification from the quantitative approach. (i) up-regulated proteins (blue), and (ii) down-regulated proteins (pink) predicted to make more interactions comprising the 2,011 quantified proteins. Non-differentially expressed proteins (yellow), which make more interactions with the up-regulated proteins (iii), with the down-regulated proteins (iv), with other non-differentially expressed proteins (v), and with both up- and down-regulated proteins (vi). Click here for file

Additional file 10 **Figure S3 Interaction network involving up and down regulated proteins in *N. caninum discharged*** tachyzoite - details. Down-regulated proteins are represented by nodes in pink, up-regulated proteins by nodes in blue, and non-differentially expressed proteins by nodes in yellow. The edges are displayed in black lines. Click here for file
