# Supplementary figures and images for: Unravelling the Neospora caninum secretome through the secreted fraction (ESA) and quantification of the discharged tachyzoite using high-resolution mass spectrometry-based proteomics
Source: Parasit Vectors. 2013 Nov 23;6:335. doi: 10.1186/1756-3305-6-335 (PMC4182915; doi:10.1186/1756-3305-6-335)

# *N. caninum* identified and quantified proteins involved in the inositol phosphate metabolism

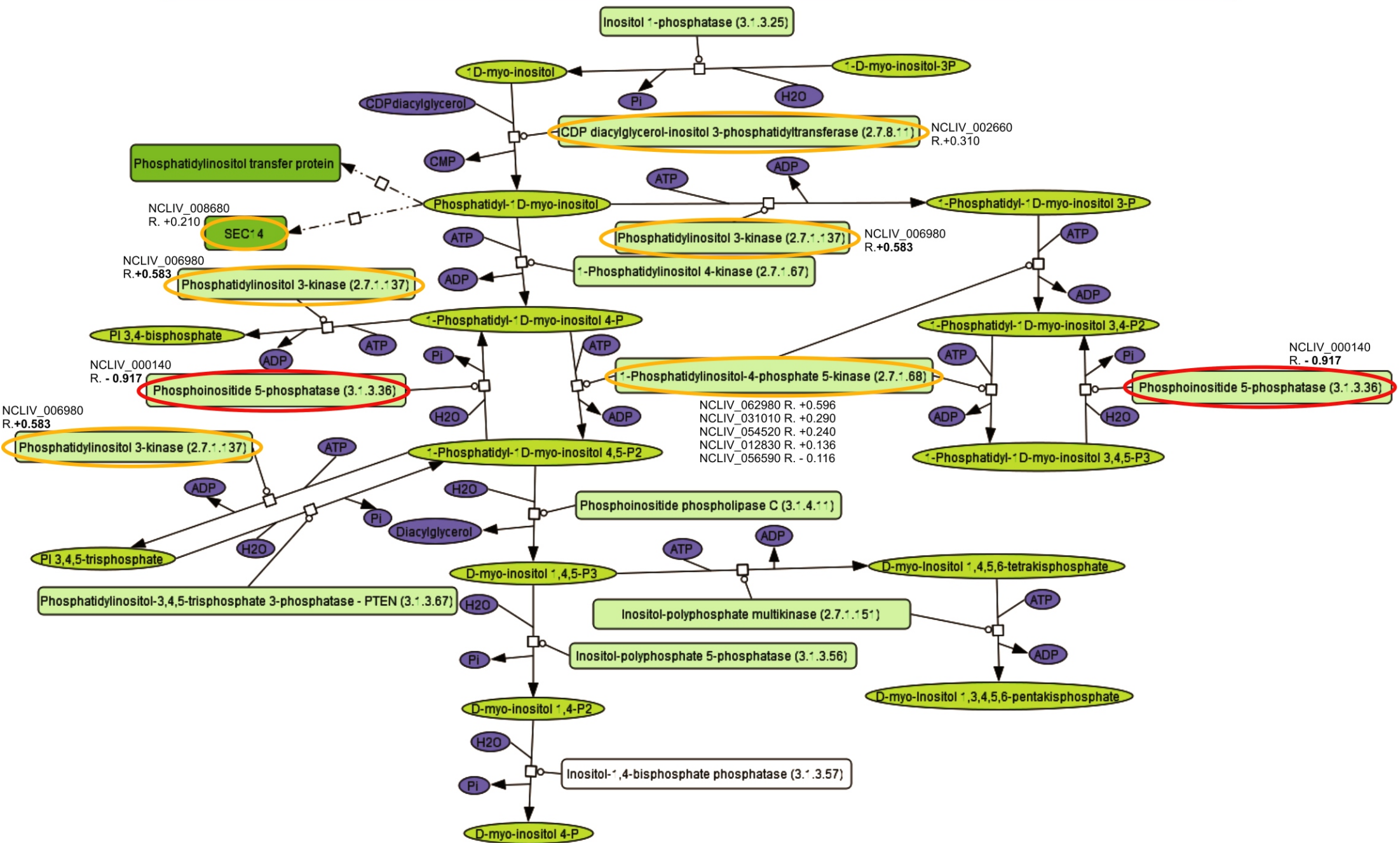

Supplement: Additional file 6 — Figure S1 Inositol phosphate metabolism adapted from LAMP (Library of Apicomplexan Metabolic Pathways). The quantified proteins in N. caninum discharged tachyzoite are surrounded by coloured circles designating their expression level (blue, up-regulated; pink, down-regulated; yellow, not differentially expressed), and their log2 ratios are also displayed. [file 1756-3305-6-335-S6.pdf]

### *N. caninum* identified and quantified proteins involved in the purine metabolism

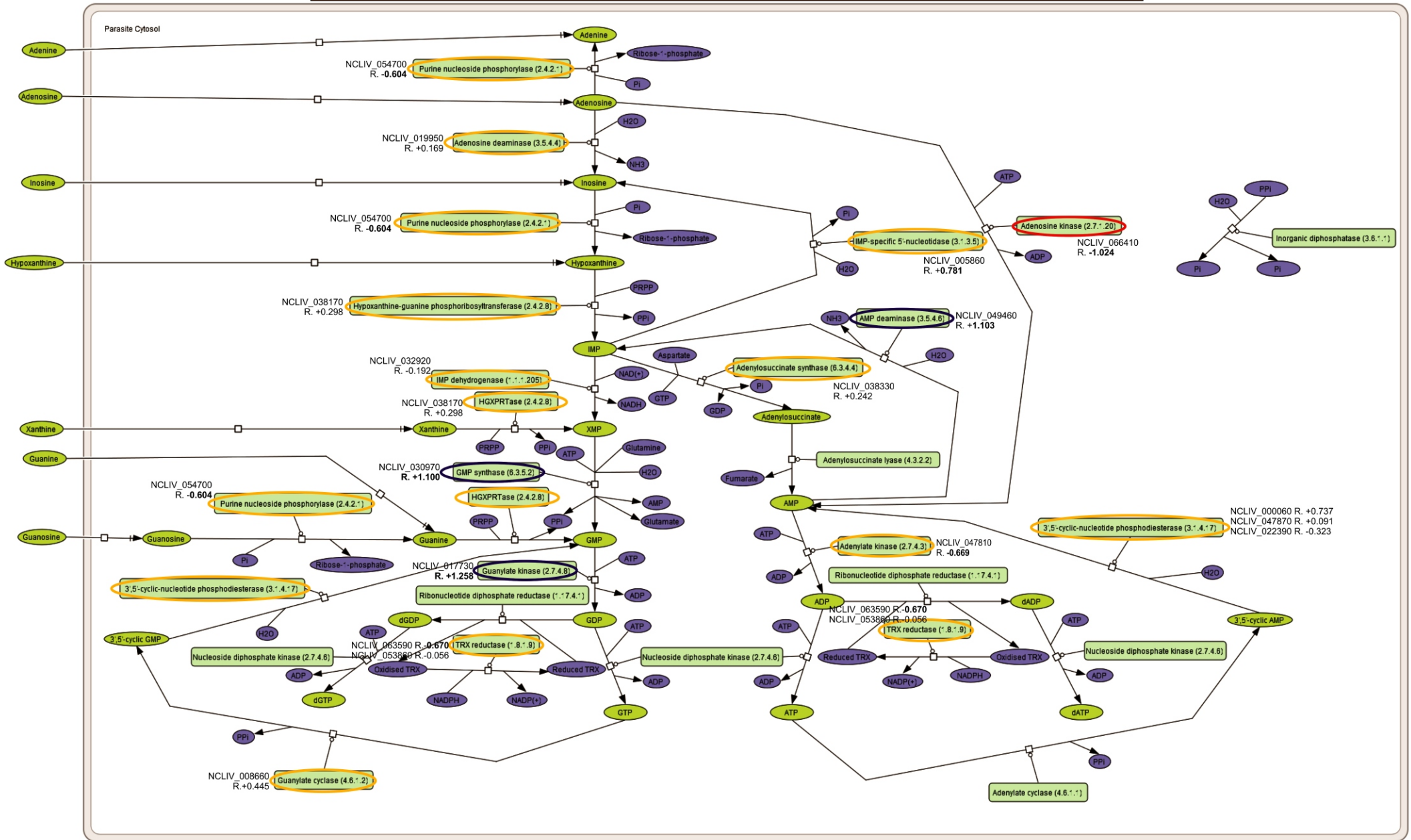

Supplement: Additional file 7 — Figure S2 Purine metabolism adapted from LAMP (Library of Apicomplexan Metabolic Pathways). The quantified proteins in N. caninum discharged tachyzoite are surrounded by coloured circles designating their expression level (blue, up-regulated; pink, down-regulated; yellow, not differentially expressed), and their log2 ratios are also displayed. [file 1756-3305-6-335-S7.pdf]

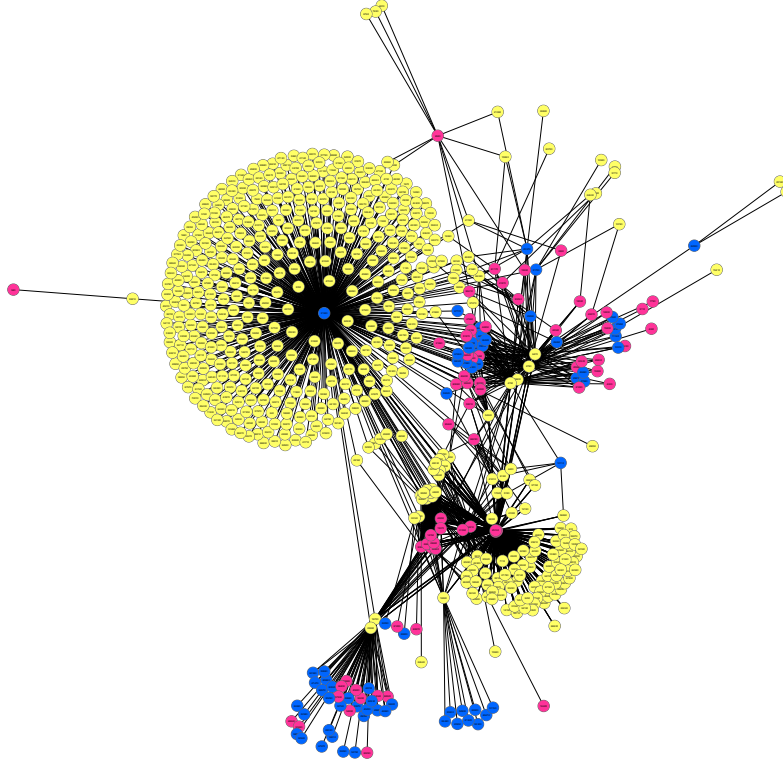

Supplement: Additional file 10 — Figure S3 Interaction network involving up and down regulated proteins in N. caninum discharged tachyzoite - details. Down-regulated proteins are represented by nodes in pink, up-regulated proteins by nodes in blue, and non-differentially expressed proteins by nodes in yellow. The edges are displayed in black lines. [file 1756-3305-6-335-S10.pdf]
